# Barriers and enablers to skin-to-skin contact at birth in healthy neonates - a qualitative study

**DOI:** 10.1186/s12887-018-1033-y

**Published:** 2018-02-09

**Authors:** Amala James Alenchery, Joanne Thoppil, Carl Denis Britto, Jimena Villar de Onis, Lavina Fernandez, P. N. Suman Rao

**Affiliations:** 1 Undergraduates, St. John’s Medical College Hospital, Bangalore, India; 2 Social Scientist, St. John’s Medical College Hospital, Bangalore, India; 3 Department of Neonatology, St. John’s Medical College Hospital, Sarjapur Road, Koramangala, Bangalore, 560034 India; 4 Maternal Health Research Coordinator, Compañeros En Salud, Chiapas, Mexico

**Keywords:** Qualitative study, Barriers, Skin to skin contact at birth

## Abstract

**Background:**

Skin to skin contact (SSC) at birth is the standard of care for newborns without risk factors. However, implementation of SSC at birth has been far from optimal. A qualitative study was undertaken to determine the barriers, enablers and potential solutions to implementation of SSC at birth in healthy newborn infants in a level III neonatal-care facility in Bangalore, India.

**Methods:**

Consultants and residents/postgraduates (PG) from the departments of Obstetrics (*n* = 19) and Pediatrics (*n* = 14) and nurses (*n* = 8) in the labor room (LR) participated in the study. In depth interviews (IDI) and focus group discussions (FGD) were carried out with an interview guide and a moderators’ guide containing inbuilt probes. Subjects of FGD were homogenous. All IDI and FGD were audio-taped, transcribed and analyzed using N VIVO version 9 (using free and tree nodes). Two authors separately coded the transcripts. Major and minor themes were identified. Rigor was ensured by triangulation and theoretical saturation. Informed consent and ethical approval was obtained.

**Results:**

All subjects were aware of SSC at birth, some of its benefits and had practiced SSC. The major barriers identified were lack of personnel (nurses), time constraint, difficulty in deciding on eligibility for SSC, safety concerns, interference with clinical routines, and interdepartmental issues. Recall of an adverse event during SSC was also a major barrier. Furthermore, we found that most participants considered 1 h as impractical; and promoted 5–15 min SSC. Minor themes were gender bias of the newborn and cultural practices.

The participants offered solutions such as assigning a helper exclusively for SSC, allowing a family member into the LR, continuing SSC after initial routines, antenatal counselling, constant reminders in the form of periodic sessions with audiovisual aids or posters in the obstetrics ward, training of new nurses and PG, and inclusion of SSC in medical and nursing curriculum.

**Conclusions:**

The major barriers to SSC at birth are lack of personnel, time constraint and safety concerns. Training, designated health personnel for SSC and teamwork are the key interventions likely to improve SSC at birth.

**Electronic supplementary material:**

The online version of this article (10.1186/s12887-018-1033-y) contains supplementary material, which is available to authorized users.

## Background

The transition from intrauterine to extra uterine life represents one of the most dynamic and potentially dangerous events in the human life cycle. This early “sensitive period” after birth during which the newborn adapts to the new world outside the mother’s womb requires intimate contact between the infant and mother in order to build a platform for bonding and to improve the physiologic and neurologic development of the child as well as make the mother more confident in her own abilities to nurse her child [1]. Skin to skin contact (SSC) at birth is the placing of the naked newly born baby prone on the mother’s bare chest at birth or soon afterward for a minimum duration of at least 1 h. The healthy full-term human infant, placed in SSC after birth, directs himself or herself to the mother’s breast and nipple and starts to suckle by about 1 h of age [2].

Health care personnel are uniquely placed to positively influence the mother-infant interaction at birth [3]. It is recommended that healthy infants should be placed and should remain in direct SSC with their mothers immediately after delivery until the first feeding is accomplished [2]. SSC at birth has been shown to have several beneficial effects for the newly born. A meta-analysis of 38 randomized controlled trials including 3472 mother-infant dyads has shown that it improved breastfeeding duration, cardio-respiratory-metabolic stability at birth and temperature. SSC at birth reduces stress associated with birth and facilitates self-regulation. Neurobehavioral benefits and positive parenting impact are evident even after a decade, making SSC at birth the optimal method of care [2].

Despite the reported benefits, direct skin-to-skin contact after birth is not universally practiced. In fact, routines exist today, that separate the mother and her newborn infant as a common practice [4]. There are several barriers to implementation of SSC at birth, even as a component of KMC at the institutional level, health personnel level and maternal or family level [5]; most related to common practice rather than to a medical concern [6].

There is a lack of qualitative studies looking specifically at early SSC, and particularly a dearth of studies exploring the perception of health personnel about SSC at birth from lower-middle income countries (LMICs). Though studies have explored barriers and enablers in the implementation of kangaroo mother care (KMC), this data from KMC implementation may not be applicable to SSC at birth as KMC [7] refers to a standardized method of care of preterm/ low birth weight (LBW) with early, prolonged SSC, frequent breast feeding and early discharge and follow-up [8] whereas SSC is defined as placing the naked newborn prone on the mother’s chest and abdomen immediately after birth [2]. 

A questionnaire based study to understand the barriers for implementation of SSC at birth showed that primary resistance to SSC at birth is at the health care worker level [9]. Furthermore, we hypothesize that most perinatal health care workers are not fully aware of the methodology and guidelines of SSC. This qualitative study is therefore undertaken to explore the barriers, enablers and solutions to promotion of SSC at birth as perceived by the health care personnel.

## Methods

### Study design

With the aim of understanding the essence of the health workers’ experience of the event of “SSC at birth”, we undertook the phenomenological life world approach of qualitative research as developed by Husserl and Merleau-Ponty [10], specifically through the use of in-depth interview and focus group discussions. The life world is the world of everyday experience, which is unique for every person, although shared with others. Phenomenological research focuses on experiences in everyday life, as experienced before theorizing, and is a well suited basis to describe and understand human experiences of a specific phenomenon such as health workers’ experiences of SSC. It is a meaning oriented approach and includes discovering, analyzing, clarifying and seeking patterns of a certain phenomenon, based on a description of how the life world of humans is experienced, acted out and described. In an interview study this means that the researcher must meet the informants and their experiences in an unprejudiced way and with a reflective, or even a self-reflective attitude.

The study was designed around the work experiences of the pediatric and obstetric staff, as in the Indian context, where SSC falls under the responsibility of the pediatrician and nursing staff with the obstetrician playing some role. Therefore, describing the lived experience of the health workers from nursing, pediatric and obstetric specialties with regard to SSC at birth, we aspired to comprehend the barriers and enablers to the phenomenon of “skin to skin contact at birth”.

### Setting

The study was conducted in the St. John’s Medical College Hospital (Bangalore, India), a tertiary care private medical college hospital, possessing a level III neonatal care facility with an average of 2500 deliveries per year. The hospital provides service to patients from all walks of life, with the motto to help serve the under-privileged societies of the nation. The hospital mainly caters to patients belonging to the lower socio-economic strata and serves as a referral center to smaller rural health-care establishments in surrounding areas, particularly in-utero referrals of high-risk pregnancies. SSC at birth was initiated in the hospital as part of routine care for the healthy newly born in January 2011. Currently, SSC is implemented during daytime only for an average period of about 10 min in the immediate postpartum period.

### Participants

The participants included 41 health care workers stationed in the labor room of the hospital, all of whom were contacted in person and agreed to participate in the study. The groups for focus group discussion were homogenous and included personnel belonging to the same category. The health personnel were categorized as: Obstetric consultants (*n* = 5) and Obstetric residents (*n* = 14), Neonatology consultants (*n* = 2), Pediatric residents (*n* = 12) and nurses of the obstetric ward and labor room (*n* = 8). Purposive sampling was conducted from each of these homogenous groups as we had direct access to all health workers in the unit involved in conducting deliveries and administering perinatal care. This was done until no new ideas emerged and we reached data saturation and triangulation. Saturation refers to a state of data redundancy where no new ideas emerge and marks the end point of the interview or discussion and triangulation is a technique in qualitative research which involves convergence of information from different sources in order to obtain different dimensions for the same phenomenon and to validate the consistency of data obtained.

### Procedure

An interview guide (Additional file 1: Annexure 1) and a moderators’ guide for focus group discussions (FGD) (Additional file 2: Annexure 2), both developed by an iterative process where a questionnaire was administered to several experts (doctors with experience in the field of perinatal research and social scientists) on the particular subject matter, who remained external to the study. Their suggestions were incorporated in the questionnaire. The questionnaire was pilot tested in the first five interviews and redundant items were deleted. This reformulated questionnaire was used to explore the barriers and enablers of SSC at birth as perceived by health personnel. Open ended questions and probes for discussion were inbuilt into the guide for a thorough understanding of the topic. The questionnaires used for the interviews and focus group discussions have been attached as copies.

In depth interviews and focus group discussions were conducted so that the individual and group perspectives could be assessed, after obtaining ethical clearance from the Institutional Ethics Board. Individual interviews were conducted with all senior consultants of the departments, while the more junior faculty - residents, fellows and junior staff nurses - were included in both interviews and focus group discussions in order to encourage emerging ideas, maintain confidentiality and to prevent the hierarchy within departments from skewing the results. Some of the junior faculty were also interviewed individually in order to enhance the quality of data obtained.

In total, 21 face to face in depth interviews (IDI) were conducted by two investigators (AJ and JT) in the NICU and the postnatal wards of the hospital (13 IDI with obstetric personnel, 7 with pediatric personnel and 1 with a labor room nurse in charge. Care was taken to ensure privacy and complete confidentiality. Informed consent was obtained prior to the interview from all subjects. The interviews were audio taped with an average duration of interviews lasting 30 min. Clarifications were sought if any aspect of the transcript was not clear.

The two investigators conducted 4 FGDs with key stakeholders – obstetric residents (*n* = 7), pediatric residents (*n* = 7), neonatal fellows (*n* = 4) and nursing staff (*n* = 7), with the help of the moderator’s focus group guide. The subjects for the FGD were homogenous and from the same category. The group discussions were held in a classroom outside the NICU and in the OBG wards classroom. At the time of the FGD, all subjects were given the informed consent form. A co-investigator, a trained social scientist, with experience in conducting FGDs assisted and plotted the sociogram, which gave a visual representation of the dynamics of the group discussion, in order to avoid any dominant or passive members in the same group and to ensure maximal and equal participation from all subjects. Multiple viewpoints and responses were discussed during the FGDs. The FGDs were audio taped. Each discussion lasted approximately 45 min. Clarifications about some aspects of the transcript were done after discussion with the participants.

Of the 41 subjects approached during the study, 17 subjects were included only in IDIs while 20 subjects were included only in the FGDs as part of four separate homogenous groups and there were 4 subjects who were included in both the IDIs and FGDs,

The researchers who interviewed the participants and facilitated the group discussions were independent medical students and did not hold any managerial role or position of authority to reduce any potential bias. The interviews and FGDs were conducted in English, which is the language of communication in this hospital, transcribed verbatim, and anonymized.

### Data analysis

The main methodology covered was a deductive analysis based on preliminary analysis and iterative reading of the data. This involved constantly comparing newly emerging themes from the data with a pre-established framework.

A framework analytical approach was used for data analysis. The steps of this analysis were as follows:

1. Familiarization with data and coding
2. Identifying a thematic framework
3. Sorting quotes
4. Placing quotes under the thematic category
5. Mapping and interpreting

Two authors (AJ, JT) initially familiarized themselves with the data by reading and rereading the transcripts which ensured an ease of accessibility to the transcript at a later stage. Then, the focus group discussions and interviews were independently coded to ensure credibility and trustworthiness. Any differences in the coding were resolved by consensual agreement of all authors. This process of familiarization and coding helped to identify new themes and categories that led to an iterative process. Several quotes in the transcripts could be identified with multiple themes and the process was reiterated in order to avoid overlooking any emerging themes [11].

N Vivo version 9 (QSR International, Burlington, Massachusetts) was used to classify the nodes as free and tree nodes. Tree nodes refer to codes which are organised hierarchically into categories and subcategories, while free nodes are emergent themes which are not attached to a pre-existing tree node [12].The nodes developed were used to code the transcripts inductively following a process of constant comparison between the emerging themes/codes and pre-existing codes. Any emergent code was added as free node or attached to a tree node according to its place in the initial thematic framework. Salient quotes were noted. Coding density, which refers to the strength of association between themes was used to identify recurrent themes. The themes with highest coding density were categorized as major themes and the others as minor themes. Based on this, a final thematic model was developed.

## Results

Based on the interviews and group discussions, several main barriers emerged, all of which were believed to prevent SSC from being carried out as routine practice for all healthy term neonates. Along with the barriers and enablers, solutions that can help promote SSC were also brought to light. The participants of the study were personnel of the obstetric, pediatric and nursing staff, comprising of males (*n* = 8) and females (*n* = 33), ranging in ages from 24 to 50 years old with the average years of experience being of 15.1 years for the consultants of both departments and 3.2 years for the residents of obstetric department and 3.5 years for the residents of the paediatric and neonatology department.

The importance attributed to SSC among the various departments, the knowledge regarding practice and need for SSC and personal experience of the staff were the foundation for the barriers to SSC at birth, and were similar among the health personnel groups, each focussing on a different subcategory.

The enablers are the existing practices which will help to further establish the practice of SSC, while solutions are merely proposed suggestions which can be utilised by the respective departments in order to promote SSC and furthermore practice it as an established standard of care.

Table 1 shows the main barriers challenging the practice of SSC, enablers that promote the practice and solutions to the perceived barriers.

**Table 1:** Barriers, Enablers to SSC and Solutions to promote SSC

Barriers	Enablers	Solutions
Skewed health-care staff client ratio in the labor room • Lack of Personnel • Time Constraints	Knowledge of Benefits • Motivation by Pediatricians	Dedicated bystander • Dedicated staff • Relative/family member
Apprehensions related to the procedure • Concern of Safety • Dilemma in decision making		Structured periodic inter-department teaching • Training staff • Reinforcement by Demonstration • Interdepartmental dialogue
Parochialism towards SSC • Lack of Awareness • General lack of belief • Lack of constant motivation	Maternal Acceptance	Antenatal awareness • Creating demand
Interdepartmental Issues Minor barriers • Interference with other clinical procedures • Gender preference for the newborn	Positive Experience	Early SSC – a practical alternative

**Fig. 1:**
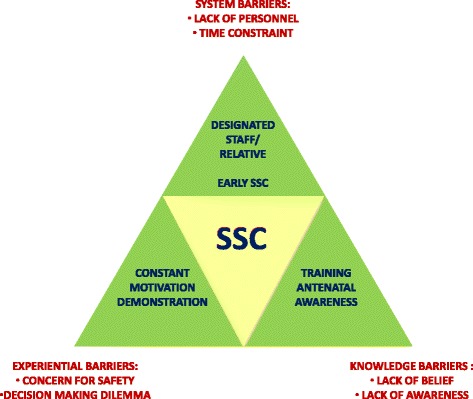
Barriers and solutions to skin to skin contact at birth

The figure serves as a graphical summary of the results and serves as a consolidated representation of the barriers to SSC and possible solutions for the same (Fig. 1).

Attempts were made to understand the nature of the perceived difficulties in implementing SSC as a routine practice and the results are as follows.

A. BARRIERS

Lack of personnel and time constraints owing to the skewed health-care staff to patient ratio were recurring barriers that emerged from all the FGDs and IDIs. Other barriers identified include concerns over the safety of the newborn falling down from the mother’s chest, lack of awareness, and doubts in the efficacy of this practice coupled with inadequate motivation to carry out SSC. Interdepartmental disagreements and dilemmas in the eligibility of newborns to undergo SSC were other barriers.

1. Skewed health-care staff- client ratio in the labor room

This was a prominent barrier during the night shifts when nurses in the labour room are especially understaffed and overworked by their wide range of duties forcing SSC to be a relatively unimportant priority, particularly when the recommended time for SCC is for an hour. The pediatrician on call has to assess the newborn in addition to a host of other responsibilities in the ward, neonatal care unit and neonatal emergency. This arrangement leads to time constraints making it very unlikely for SSC to be practiced outside regular working hours i.e. 9 am to 4 pm. It is not uncommon for more than one delivery to occur simultaneously in this setting, further stretching resources and making SSC in a situation like this a near impossible task.

*N: “Mainly it is the lack of staff. For this one-hour, we can’t keep one staff throughout*.”

*Ped: “The pediatricians on duty, being understaffed, have to leave their designated ward duty and attend to the deliveries. Hence, staying with the baby for the entire duration of SSC is not feasible especially when the patient influx is high”.*

*Ped:* “*Of late, the issues we’ve had are usually the nurses are in a hurry to finish their work and get done with the delivery, either because they have got more deliveries coming in or they’ve got admissions coming in or they’re short of staff*.”

A.2. Apprehensions related to the procedure

A major barrier perceived during the course of the study was recall of an adverse event in the labor room wherein a healthy baby became cyanosed during SSC and required resuscitation. The obstetricians felt that as the treating physician, they would be held accountable if there was any untoward event.

*Ob-gyn*: “*We are answerable more than the pediatrician. For 9 months, the patient has seen us, so they’ll expect more from us. So it is more of our responsibility. That’s why we are a little jittery with care that nothing should happen to the baby and mother*.”

This was coupled with other concerns like the baby slipping from the mother’s chest and falling as well as being uncomfortable making the decision regarding a newborn’s eligibility to undergo SSC. Most often, young pediatric residents or fellows are responsible for deciding SSC eligibility, which is not always straightforward.

*Ob-gyn*: *“Definitely there is fear of the baby falling if we’re thinking the baby should be on the mother while doing episiotomy and the mother is tossing and turning in pain.”*

*Ped:* “*Deciding on eligibility for SSC is a grey zone*”

A.3. Parochialism towards SSC

In a teaching hospital, there is a constant turnover of staff. New staff is not always adequately sensitized to the importance and benefits of SSC, and in this study most of the members of the obstetric department were not aware of the guidelines. In fact, they felt implementing SSC for one whole hour immediately after birth was almost impossible and were unwilling to even consider the possibility. Few even considered it more harmful than beneficial. Several other doctors, especially the new resident doctors felt the same, as they were unaware of the practice and its benefits. Disinterest and general lack of buy-in was a barrier, which seemed to be widespread among all departments. More than logistic issues, the attitude of health care personnel to the process emerged as a barrier.

*Ob-gyn*: “*Nobody has really told us how it is to be done. We just know that SSC is good for the baby and its being done. But how it is to be done and what is to be done, nobody has really told us. Why it is to be done, how it is beneficial to the mother or baby, no one has taken that extra mile to tell it to us, the gynecologists. Maybe the people doing it will know about it but they have not told us why.”*

*Ped:* “*I think the biggest thing is not in lack of personnel, I think it’s in the attitude of people; wherein people don’t see the need or they don’t see the importance of SSC*.”

Like any other practice that is not a mandated protocol, frequent motivation is essential to ensure that SSC is practiced as a routine procedure. Most of the participants in the study felt that since it was a relatively new practice, it was difficult to remember to promote SSC and it was the responsibility of the pediatricians to be a constant source of motivation.

A nurse on labor room duty expressed that the paediatrician attending the delivery was hastening them to bring the baby away from the mother for routine examination as they had to tend to other sick infants and could not spare one hour of their time solely for SSC. To quote her:

*N: “They are busy, so bring the baby, they’ll say. They want to collect samples. They say that you can do after we go. Sometimes they do not know we are giving SSC.”*

A.4. Departmental issues

The nature of SSC as such calls for teamwork and close inter-departmental co-operation. Obstetricians and nurses perceived the onus of SSC to lie on the pediatricians. However, the nurses felt the pediatricians were hastening the shift of the baby from the mother to the resuscitation station, while the pediatricians felt the obstetricians were more inclined to separate the baby from the mother and the obstetricians state their main concern is to ensure progression and completion of labor with no complications and hence, do not concentrate on SSC.

Interestingly, the head nurse in the labor room felt that interdepartmental issues were one of the most addressable barriers to facilitating and promoting SSC by ensuring continuing conversation among the members of the departments.

*N: “We cannot identify the staff and say it is your responsibility. If we say that and identify, it is not going to happen smoothly. It is the teamwork; equally the paediatrician, gynaecologist and labour room sister are responsible. Suppose if one of them is not doing it the other person has to remind.”*

A few minor barriers that were also of concern were the lack of personal experience by the staff, interference with clinical routines, for example, during the period when the shift change of staff occurs, they tend to concentrate on their routine work rather than spending time on establishing and maintaining SSC, and the lack of willingness from the mother. Another minor barrier which emerged was the maternal refusal for SSC due togender preference, which refers to the prevailing preference for the birth a male child by the families in the setting of a hospital in India, a low middle income country (LMIC).

N: “*They [the mothers] are expecting a boy baby and they get a girl. So they push the baby away.”*

B. Enablers for SSC

Though there were many constraints that challenged the practice, the factors that motivate the implementation of the practice were:

1. Knowledge of benefits of SSC

Knowledge of the benefits of SSC mainly by the nurses and pediatricians was one of the key factors motivating health personnel to implement SSC. They believed that it would actually make a difference. The main source of knowledge regarding SSC was either from the nursing curriculum or from the teachings of a dedicated and motivated paediatrician.

*N: “First of all it helps in bonding, then baby’s temperature, feeding issues, psychological effects on the mother as well as the baby. It’s an opportunity for the mother and baby to bond. It needs to be done.”*

Motivation by paediatricians

The nurses also stated that the implementation of SSC was to a large extent dependent on the pediatrician attending the labor call. A very motivated pediatric resident would ensure SSC implementation and would even teach the nurses, because they have witnessed the benefits of SSC.

*Ped: “We are keen on doing SSC so we are constantly motivating them (nurses).”*

*N: “The pediatricians are generally around to remind us to do that.”*

B.2. Positive experiences

A firsthand experience of the benefits was crucial. Obstetricians, pediatricians and nurses who had practiced SSC and had firsthand experience of the benefits were the biggest promoters of SSC.

*Ob-gyn*: *“Yeah, definitely, there is lot of decreased crying, decreased pain for the mother. The mother is not really concerned about her episiotomy and suturing.”*

*N: “The child will be crying and when we put the child on the mother’s breast, somehow I’ve seen how the child stops crying and you know, he feels more comfortable when he’s on the mother.”*

B.3. Maternal acceptance

The level of acceptance of the practice by the mothers and their families were equal and positive despite difference in educational, cultural and religious backgrounds, as perceived by the medical personnel during the interviews and FGDs. Even if some mothers were apprehensive to begin with, their fears were quickly allayed once a nurse held the baby in place.

Hygiene issues of placing a newborn on the mother’s bare chest in the labor room were of least concern as most of the doctors and staff believes the exchange of commensal skin flora between the mother and the newborn was beneficial.

C. Solutions

1. Dedicated bystander

Even amongst those motivated and aware of the benefits, it was perceived by health personnel across the specialties that a dedicated person for ensuring the safety of the baby would ensure SSC implementation. The dedicated person for ensuring SSC need not be a qualified staff nurse; it could be a relative who holds the baby in SSC.

*N: “If one person is given the responsibility of taking care of the SSC, then I think it is possible that we can do (it) for most of the patients. Then in night deliveries, where we are not able to do now, we can do it.”*

*Ob-gyn: “Maybe a responsible attender could be let in like her mother so that they can do the SSC. I mean we are suturing the episiotomy at one end and they can stand in the other end and hold the baby.”*

C.2. Structured, periodic inter-department teaching

Training staff, subsequent reinforcement by periodic demonstration and consolidation by constant constructive interdepartmental dialogue are imperative to ensure the sustainability of this practice. Considering the high turnover of staff in the institute, ongoing training of nurses is needed. Training of student nurses was perceived as one way of improving the practice. Even the more experienced staff felt that they had to undergo the experience of actually providing SSC in order to subsequently advocate its practice. Revising the guidelines and holding demonstrations in a collaborative manner is necessary to motivate staff and promote adherence to SSC. A team approach where all stakeholders are perceived as equal partners was thought to be a sufficiently inclusive solution.

*N: “Whoever has joined newly to OB ward… whoever is assisting in deliveries definitely need to be educated. I think the education can start from all the nursing students who come there.”*

*Ped: “I don’t think you can make somebody to believe just by teaching I think they have to see themselves to actually believe in skin to skin contact, so if they see those kind of things with their own eyes I think it will be better rather than teaching them.”*

*N: “If we want to include SSC in our hospital, then I think that the head of neonatology should speak to the head of OBG. We should have a meeting with the unit chiefs, all staff and pgs. This can be easily implemented because there are no costs.”*

C.3. Antenatal awareness – creating demand

Creating demand by increasing the awareness among the mothers by antenatal education was offered as one solution for improving SSC implementation.

*Ob-gyn: “I think posters or written material, any audio-visual aid, definitely will help. And even maybe audio-visual aids for the family in the OB ward, maybe a video where they will be shown what will be done, if they don’t have time to go to each of the mothers and counsel them, so that they’re more aware and sometimes, at least some patients ask questions and the demand from the patients will also increase.”*

C.4. Early SSC – A practical alternative for SSC at birth

To overcome their inability to provide 1 h SSC at birth, the health personnel offered the solution of “Early SSC” as opposed to not implementing SSC.

*Ob-gyn: “Immediate Skin to Skin is a little difficult to practice but probably after the baby is stabilized and mother is back on the bed, probably EARLY SSC for 1 hour may not be that difficult. Mother is also comfortable in her bed, at that point of time to give the baby SSC for one hour may be a lot easier for the doctors and the staff. Even the sisters, the staff nurse will be happy to do that.”*

## Discussion

SSC at birth though recommended for all newborns who do not need resuscitation at birth, is not routinely practiced in most settings across India. Implementation of SSC at birth in the dynamic delivery room needs a concentrated combined effort from doctors across the different specialties of obstetrics and pediatrics and the labor room nurses who strive towards ensuring labor is safe for the mother and the baby. The ultimate decision as to whether SSC is to be implemented is predominantly made by the pediatricians as the obstetricians are pre-occupied with the welfare of the mother to ensure safe labor process.

The barriers elicited in this study can be grouped under three main factors, namely time constraints (an opportunity barrier), parochialism towards the process (a motivation barrier), and a general lack of buy-in (a motivation barrier),that make it difficult for obstetric staff to improve their clinical behavior as defined by the World Health Organization’s Safe Childbirth Checklist Implementation Guide [13].These barriers are described as opportunity barriers, which refers to the “environmental or contextual factors beyond an individual’s control (for example: leadership support challenges, human resource, time or supply constraints)”, motivation barriers, which refers to a lack of “interest or internal belief” in the procedure, and ability barriers, which refers to a lack of “skill, knowledge, or technical confidence.” [14].

This can in turn be expressed in terms of the Behaviour Change Wheel, which is a structured approach for initiating and designing policy changes by using the COM-B (Capability, opportunity, motivation and behaviour) model. This model identifies behaviour to be a part of an ever changing system which is in constant interaction with all the interventions which influence the new policy or intervention and acts as a framework for the same [15].

Though the skewed staff-patient ratio in the labor room is an established fact in LMIC settings, little has been done to bring about a change in the system. The solutions to these seem straightforward in providing additional staff. However, in resource-limited settings, providing more trained personnel can be challenging due to budget constraints, lack of lucrative incentives and a weak retention policy. In some settings, permitting a relative in the labor room is a potential solution which is endorsed in baby friendly hospital initiative (BFHI) manual suggesting that a “family member can stay with the mother and the baby” if no staff is available to stay with mother and baby [6]. The main barrier to allowing a family member into the delivery room is that many hospitals in the region, such as ours, do not permit family members inside the labor room as part of the hospital protocol, due to lack of space and interference with routine procedures during labor and delivery. Also, most of the doctors feel that the mother is in too much pain and under this particular circumstance cannot solely be responsible to hold the baby and participate actively in SSC. However, the benefits of implementing routine SSC to both newborn and mother will greatly outweigh the difficulties in finding a way to accommodate an extra person in the labor room. A hospital staff – a helper /aide (less trained than a nurse) could be another option and could also be used for quality control in addition to ensuring adherence to guidelines. The BFHI manual suggests that **“**if the delivery room is busy, the mother and baby can be transferred to the ward in skin-to-skin contact, and contact can continue in the ward”. This gives rise to a concept of “early SSC” rather than immediate SSC which aims to overcome the barriers of time and personnel constrains as a family member, who is usually present to take care of the mother and baby, can be delegated this responsibility.

Awareness and belief in the practice of SSC at birth came up as both a barrier and an enabler. Within all the limitations and restrictions of the system, the overwhelming belief in the practice is one the key enablers of the practice.

Implementation of potential solutions will only be successful if there is an understanding of the manifold benefits of SSC as well as by actual practice and demonstration of SSC. The awareness needs to be created both in the provider and amongst the patients. From the patient perspective, this should be initiated during antenatal visits when expectant mothers are more receptive to understanding the numerous benefits. These behavioral changes require effective communication aids such as audiovisual aids and antenatal counselling. For the health care worker perspective, “seeing is believing”. Learn, Do and Teach would be a good model to adopt as most personnel who have perceived the benefits of SSC such as reduction in pain during episiotomy, consider having had personal experience as one the most important enablers of SSC practice. This model is an extension of the study that looked at two techniques- immersion and education for improving the practice of SSC among health care providers, which concluded that immersion techniques, wherein the participants were educated about the procedure or intervention and being experts in the matter were closely monitored for evolving behavioural changes to accommodate the new technique, had better response towards establishing sustainable practice of SSC when compared to education alone [14].

A collaborative concentrated effort by all concerned personnel is needed for successful implementation since care at birth is a multidisciplinary responsibility. It is important that key stakeholders such as senior obstetricians recognize the importance of SSC at birth. Their concern of safety is reasonable as SSC at birth, like any other medical procedure, is not without a chance of rare adverse events. Safety of the newborn is paramount and reports of apnea and hypoxic brain injury following SSC are known [16]. A single adverse event could convert the personnel to a total non-believer and a threat to the implementation of SSC. There are two issues regarding safety – the first is correct identification of the newborn eligible for SSC at birth and the second, continued monitoring of the baby during SSC. Training all health personnel in the basic steps of neonatal resuscitation would empower them to make the correct decision at birth and would address the first issue; which also came up as a barrier for implementation of SSC. Having a dedicated person to ensure adequate monitoring and safety would overcome the fears expressed by the obstetricians. Further interdepartmental discussions and joint sessions on improving awareness would help in reaching the common goal. In a hierarchical environment such as a medical school, having the senior professors convinced would have a percolating effect on the rest of the department with regard to implementing and adhering to SSC. Consistent motivation by the senior obstetricians, pediatricians and nurses is needed for the execution of this evidence-based practice. Though it is important that the different departments work together, it is perceived that the onus of SSC promotion rests with the pediatrician. Incorporating the practice and benefits of SSC into the medical and nursing curriculum will be an effective way to sensitize the next generation of perinatal professionals to this practice and will act as a long-term solution.

Not surprisingly, the common barriers identified were similar to the barriers identified in a recent systematic review to implementation of Kangaroo mother care in preterm babies [7] which included “Actual increased workload due to KMC”, “concerns about medical conditions/care”, “Lack of clear guidelines / training”, “general lack of buy-in/ belief in efficacy” and “belief that it causes extra work”. Another top barrier specific to LMIC was “issues to facility, environment and resources” which referred to the lack of adequate resources as perceived by the mothers. “Support from family, friends, and other mothers” was the top enabler of KMC in the same study and can probably be extrapolated to SSC at birth [7]. Involvement of the grandmother has been postulated as an effective means of promoting KMC at home in African countries [17] and can also be done for SSC. The emotional support offered by families is an important and crucial enabler of practice [18]. “Support from staff or community health workers” was the fourth-highest- ranked enabler for practice in the systematic review but when only LMIC publications were considered, it was only the 7th ranked enabler highlighting that staff support for KMC not only plays a crucial role but also that it needs further strengthening in LMIC. These are readily applicable for SSC at birth.

### Limitations of the study

One of the limitations of the study has been that the focus group discussion has been amongst homogenous groups. Labour room being a dynamic setting where the different specialties interact, FGD with participants from the 3 groups could have brought out different issues.

## Conclusions

This study provides a set of synthesized factors regarding the system, experiential and knowledge barriers to the implementation of SSC at birth. By providing potential solutions to these barriers and highlighting the enablers of SSC, the results of this study could aid program implementers, policymakers, and researchers to implement and scale up this important tool of SSC at birth that has the potential to improve breastfeeding practices.

## Additional files

Additional file 1: Interview Guide. (PDF 71 kb)

Additional file 2: Focus Group Discussion Guide. (PDF 73 kb)

## Abbreviations

BFHI: baby friendly hospital initiative
FGD: Focus group discussions
IDI: In depth interviews
KMC: Kangaroo mother care
LMIC: Low middle income countries
LR: Labor room
NICU: Neonatal intensive care unit
PG: Post graduates
SSC: Skin-to-skin contact
