# Supplementary material for: Barriers and enablers to skin-to-skin contact at birth in healthy neonates - a qualitative study
Source: BMC Pediatr. 2018 Feb 9;18:48. doi: 10.1186/s12887-018-1033-y (PMC5807736; doi:10.1186/s12887-018-1033-y)
Supplement: Supplementary file 1 — Interview Guide. (PDF 71 kb) [file 12887_2018_1033_MOESM1_ESM.pdf]

Interview Guide

Introduction:

Thank you for accepting to be interviewed by us. The study we are undertaking is to understand more about the process of skin to skin contact (SSC) post birth in healthy new born infants, born by normal vaginal delivery. I will be asking you several questions which are relevant to the study. You may respond to these queries in any way you feel comfortable. It is perfectly fine if you do not want to respond. At any point during the interview, if you are not clear about any questions, you are free to clarify the same with us and ask us to explain further. The information obtained during the interview will be kept confidential and will be shared only with the research team. We would like to audio record the interview in order to ensure that we do not miss out any salient issues. The recordings will be kept confidential. Your identity will be protected and your interview will also be labelled in codes. Is it OK with you that we audio record the interview?

Personal Information:

Name: __________ (optional)
Age: Sex:
Designation: Staff Nurse/Senior Staff Nurse/ JR/SR/ Consultant
Qualification: GNM/B Sc/M Sc/MBBS/MD/ DGO/ DCh
Months / Years of experience with newborns_________

THEME: Barriers to skin to skin contact in healthy neonates born by normal vaginal delivery as perceived by health personnel

Component- Awareness of current scenario in the hospital

Question- Are you aware of the practice of SSC in our hospital?

Probe-

1. How did you come to know about it?
2. What is routinely practiced in our hospital? Who decides when the baby is ready for SSC?
3. For how long is it practiced?
4. What are the routine checkups conducted for the neonate that you are aware of?

Component- Benefits of the practice of SSC

Question- Why do you think we should do SSC after birth and how is it helpful for the baby?

Probe-

1. What do you feel about the practice of ssc?
2. Is there any specific incident that comes to you regarding SSC? What happened? Can you elaborate a little about it?

3. Do you feel it has any effects on (temperature, blood sugar) the infant’s physiology?
4. How do you feel it effects the mother? The pain of episiotomy?
5. How do you feel the child responds? Reduced stress and crying?
6. How do you think the mother responds? Can you please elaborate..?
7. Does it have any effect on bonding between mother and infant in the initial period?
8. Does it influence breastfeeding in any manner (time of initiation and duration)?

Component- Barriers to SSC

Question- Why do you think SSC doesn’t happen all the time?

Probe-

1. Do you feel it interferes with your work? Do you feel it interferes with routine care of the mother and baby? Can you give me an example of any such incident that you remember?
2. How do the personnel involved in implementing it feel about the practice? How do you feel about this practice?
3. Were there any Perceptual fears regarding this practice? How was the situation handled?
4. Can you please tell me about an incident you can recall where it was difficult to decide when the baby is ready for SSC?
5. Do you feel there is Lack of time/ lack of personnel?
6. Who decides whether or not a neonate is ready for SSC? Does this have any effect on the proper implementation of the practice? In what manner?
7. Has any concern arisen regarding the safety of the baby? Can you please elaborate?
8. Have you been concerned of hygiene during SSC?
9. Do you think the clothes of the mother hinders/ helps with SSC?
10. How do the mothers respond to it? Have the mothers expressed any concern regarding safety of the baby? (Mothers chest isn’t sterile)
11. Have the mothers refused ssc for any reason? How did you handle the situation? (inappropriately dressed)
12. Do you feel any religious or cultural group responds better to the practice? How do different groups respond to the practice?
13. Is it followed correctly? Doctors not promoting SSC?
14. How practical do you think the practice is in all situations? Does it interfere with care of baby and mother post birth?
15. I’m not sure I understand this, would you care to explain that to me..?

Component- Methods to overcome

Question- How do you think these barriers preventing routine SSC practice can be overcome?

Probe-

1. Creating more awareness regarding practice and its benefits?
2. How will educating the new staff impact the process?
3. Will conducting sessions on the same and putting Posters in the NICU/ ward/ Labor room affect the process?
4. How do you feel the process will be helped if more personnel were available?
5. Do you think the mother’s chest needs to be cleaned prior to SSC?

6. Do you feel there is anything else we can do? Can you please elaborate?
7. How do you feel about educating mothers about the benefits in order ensure proper practice? Do you think antenatal counseling of mother/family regarding the practice is required? have you had any difficult experiences in the same area?
