# Supplementary material for: Barriers and enablers to skin-to-skin contact at birth in healthy neonates - a qualitative study
Source: BMC Pediatr. 2018 Feb 9;18:48. doi: 10.1186/s12887-018-1033-y (PMC5807736; doi:10.1186/s12887-018-1033-y)
Supplement: Supplementary file 2 — Focus Group Discussion Guide. (PDF 73 kb) [file 12887_2018_1033_MOESM2_ESM.pdf]

Focus Group Discussion Guide

Introduction:

Thank you for accepting to participate in the focus group discussion by us. The study we are undertaking is to understand the barriers perceived by the health care personnel in the implementation of skin to skin contact (SSC) post birth in healthy new born infants, born by normal vaginal delivery and to try and overcome the same.

We will have a discussion on the various barriers that hinder the implementation of skin to skin contact (SSC) post birth in healthy new born infants, born by normal vaginal delivery. You may respond to these queries in any way you feel comfortable. It is perfectly fine if you do not want to respond. At any point during the discussion, if you are not clear about any questions, you are free to clarify the same with us and ask us to explain further. The information obtained during the group discussion will be kept confidential and will be shared only with the research team. We would like to audio record the interview in order to ensure that we do not miss out any salient issues. The recordings will be kept confidential. Is it OK with you that we audio record this discussion?

Respondent register:

Date: Place: Time:

Name of moderator:

Name of note taker:

No	Name	Age	Gender	Details of participants

THEME: Barriers to skin to skin contact in healthy neonates born by normal vaginal delivery

Component- Awareness of current scenario in the hospital

Question- Are you all aware of the practice of SSC in our hospital?

Probe-

1. How did you come to know about it?
2. What is routinely practiced in our hospital? Who decides when the baby is ready for SSC?
3. For how long is it practiced?
4. Are you aware of the routine checkups conducted for the neonate?

Component- Benefits of the practice of SSC

Question- Why do you think we should do SSC after birth and how is it helpful for the baby?

Probe-

1. Is it required? Do you feel it is required?
2. Is there any specific incident that comes to your mind regarding SSC?
3. Any other effect..? Effects on temperature, blood sugar, infant physiology?
4. Reduced crying of infant? Reduced stress and crying?
5. Effects on the mother? Is there improved bonding between the mother and child?
6. Is there a positive outcome on breastfeeding in terms of time of initiation and duration?

Component- Barriers to SSC

Question- Why do you think SSC doesn't happen all the time?

Probe-

1. Any interference to routine care of mother and baby?
2. Do the personnel involved believe in it? Do you believe in this practice?
3. Perceptual fears
4. Difficult to decide when the baby is ready for SSC..?
5. Lack of time/ lack of personnel? Doctors are busy?
6. Concern for safety of the baby. Do you feel it is harmful?
7. How do the mothers respond to it? Has the mother expressed any concern regarding safety of the baby?Mothers chest isn't sterile?
8. Cultural or religious barriers?
9. Mother is shy? Inappropriately dressed?
10. Is it followed correctly? Doctors not promoting SSC?
11. Is it practical in all situations? Does it interfere with care of baby and mother post birth? Suturing?

Component- Methods to overcome

Question- Do you think it is possible to overcome these barriers preventing routine SSC practice? If yes,how?

Probe-

1. Creating more awareness regarding practice and its benefits?
2. Educating new staff?
3. Conducting sessions on the same? Posters in the NICU/ ward/ Labor room?
4. More personnel
5. Do you feel there is anything else we can do?
6. Will teaching mothers about the benefits ensure proper practice? Counseling parents and close relatives?
